# Early diagnosis of sepsis in emergency departments, time to treatment, and association with mortality: An observational study

**DOI:** 10.1371/journal.pone.0227652

**Published:** 2020-01-22

**Authors:** Gunnar Husabø, Roy M. Nilsen, Hans Flaatten, Erik Solligård, Jan C. Frich, Gunnar T. Bondevik, Geir S. Braut, Kieran Walshe, Stig Harthug, Einar Hovlid

**Affiliations:** 1 Department of Social Science, Western Norway University of Applied Sciences, Sogndal, Norway; 2 Department of Global Public Health and Primary Care, University of Bergen, Bergen, Norway; 3 Department of Health and Functioning, Western Norway University of Applied Sciences, Bergen, Norway; 4 Department of Clinical Medicine, University of Bergen, Bergen, Norway; 5 Clinic of Anaesthesia and Intensive Care, St. Olavs Hospital, Trondheim, Norway; 6 Department of Circulation and Medical Imaging and Mid-Norway Sepsis Research Group, Norwegian University of Science and Technology, Trondheim, Norway; 7 Institute of Health and Society, University of Oslo, Oslo, Norway; 8 National Centre for Emergency Primary Health Care, NORCE Norwegian Research Centre, Bergen, Norway; 9 Stavanger University Hospital, Stavanger, Norway; 10 Norwegian Board of Health Supervision, Oslo, Norway; 11 Alliance Manchester Business School, University of Manchester, Manchester, England, United Kingdom; 12 Department of Research and Development, Haukeland University Hospital, Bergen, Norway; 13 Department of Clinical Science, University of Bergen, Bergen, Norway; Vita Salute University of Milan, ITALY

## Abstract

**Background:**

Early recognition of sepsis is critical for timely initiation of treatment. The first objective of this study was to assess the timeliness of diagnostic procedures for recognizing sepsis in emergency departments. We define diagnostic procedures as tests used to help diagnose the condition of patients. The second objective was to estimate associations between diagnostic procedures and time to antibiotic treatment, and to estimate associations between time to antibiotic treatment and mortality.

**Methods:**

This observational study from 24 emergency departments in Norway included 1559 patients with infection and at least two systemic inflammatory response syndrome criteria. We estimated associations using linear and logistic regression analyses.

**Results:**

Of the study patients, 72.9% (CI 70.7–75.1) had documented triage within 15 minutes of presentation to the emergency departments, 44.9% (42.4–47.4) were examined by a physician in accordance with the triage priority, 44.4% (41.4–46.9) were adequately observed through continual monitoring of signs while in the emergency department, and 25.4% (23.2–27.7) received antibiotics within 1 hour. Delay or non-completion of these key diagnostic procedures predicted a delay of more than 2.5 hours to antibiotic treatment. Patients who received antibiotics within 1 hour had an observed 30-day all-cause mortality of 13.6% (10.1–17.1), in the timespan 2 to 3 hours after admission 5.9% (2.8–9.1), and 4 hours or later after admission 10.5% (5.7–15.3).

**Conclusions:**

Key procedures for recognizing sepsis were delayed or not completed in a substantial proportion of patients admitted to the emergency department with sepsis. Delay or non-completion of key diagnostic procedures was associated with prolonged time to treatment with antibiotics. This suggests a need for systematic improvement in the initial management of patients admitted to emergency departments with sepsis.

## Introduction

Sepsis is a major challenge, being present in a large proportion of hospitalizations that culminate in death [1–3]. Most sepsis cases seem to arise outside hospital settings [4], and these patients present to emergency departments with heterogeneous signs and symptoms, making detection and diagnosis challenging [5]. New sepsis criteria and early antibiotic treatment has been a major focus of research and debate over the last years [6] but factors associated with delayed treatment in the emergency departments have received less attention.

Previous research, mostly based on single case studies and smaller patient cohorts, suggests that systematic screening and diagnostic procedures for recognizing sepsis are not consistently carried out according to current guidelines [5, 7] and that sepsis is not recognized early enough [7]. Early recognition of sepsis is of critical importance for timely treatment [8–10], and compliance with sepsis guidelines is associated with improved outcomes [11–13]. However, no studies have assessed the association between timeliness of diagnostic procedures and time to treatment [11]. More knowledge about such associations can prove useful in improving initial care of the many patients admitted to emergency departments with sepsis. Moreover, there is a need for robust data documenting the extent to which diagnostic procedures are delayed or not carried out for patients with sepsis presenting to the emergency room.

The objectives of the study were to assess the timeliness of diagnostic procedures for recognizing sepsis in emergency departments and to evaluate associations between timeliness of procedures and time to initial administration of antibiotics and association between time to antibiotic administration and 30-day all-cause mortality.

## Methods

### Setting and participants

We conducted a multicenter, observational study based on data in electronic health records of 24 Norwegian hospitals.

The Norwegian health care system is publicly funded, and it scores relatively high on the Organisation for Economic Co-operation and Development’s quality indicators [14]. In Norway, primary care physicians decide whether to refer patients with suspected sepsis to an emergency department for further assessment and treatment.

This study is a part of a 4-year longitudinal research project to assess the effects and outcomes of inspections on early detection of sepsis and time to treatment in emergency departments. The project was initiated in 2015 by the Norwegian Board of Health Supervision, which is the body delegated with the overall responsibility for external inspections of health care in Norway. The protocol for this project has been published previously [15]. In the present article, we report the results of the first part of the study, establishing baseline levels of compliance with sepsis guidelines, and assessing the associations between delayed diagnostic procedures and time to treatment and between time to treatment and mortality.

### Measures of care delivery and outcome

The Norwegian Board of Health Supervision identified key clinical practices involved in recognition of sepsis by examining international guidelines [16, 17] and receiving advice from experts on sepsis. The choice of which care processes to include was based on key elements of the guideline: screening for sepsis and diagnosing sepsis, source control, and treatment. Operationalizing these elements into process measures of care delivery was a pragmatic decision based on what data that could be expected to be available in the electronic health records. The data were collected by inspection teams, who evaluated the practices at each hospital. We operationalized these practices into process measures of care delivery, which we used as the study variables (see Box 1). We defined mortality as all-cause mortality within 30 days from hospital admission.

**Box 1. Clinical processes of delivery of sepsis care.**

- Proportion of patients triaged within 15 minutes of arrival at an emergency department.*
- Proportion of patients assessed by a physician in accordance with the urgency specified in the initial triage.
- Proportion of patients whose vital signs were measured within 1 hour of arrival at an emergency department.
- Proportion of patients whose blood lactate was measured within 1 hour of arrival at an emergency department.
- Proportion of patients from whom blood samples^†^ were taken within 1 hour of arrival at an emergency department.
- Proportion of patients from whom blood cultures were taken before administration of antibiotics.
- Proportion of patients with adequate supplementary investigations to detect the focus of infection.
- Proportion of patients adequately observed^‡^ while in an emergency department.
- Proportion of patients who had received antibiotics within 1, 2, 4, and more than 4 hours.

* Norwegian hospitals are required to establish a system for prioritizing patients admitted to emergency departments. The scales that are in use are based on the South African Triage Scale (SATS) and the Rapid Emergency Triage and Treatment System (RETTS).

^†^ Leukocyte count, hemoglobin, C-reactive protein, creatinine, electrolytes, platelet count, glucose, bilirubin, blood lactate

^‡^ ‘Adequate’ is defined as continual observation and measurement and documentation of vital signs at least every 15 minutes in critically ill patients with sepsis and organ failure, measurement and documentation of vital signs every 15 minutes if a physician has not examined a patient with sepsis but no documented organ failure, and every 30 minutes after first examination in such patients unless the physician decides otherwise.

### Study cohort

We sampled data from electronic health records of patients admitted to 24 Norwegian hospitals from May 2015 through February 2017. Hospital size and geographic location were the main inclusion criteria. The 24 hospitals were representative of Norwegian hospitals with emergency departments and included all university and regional hospitals in Norway and a geographically based selection of local hospitals, together serving 75% of the total Norwegian population of 5 million. The hospitals ranged in size from 58 to 1640 beds and had emergency departments that served their local or regional communities.

We defined sepsis as *the suspicion of infection together with two systemic inflammatory response syndrome signs*, in accordance with internationally established and widely adopted definitions of sepsis at the time the protocol was developed [16]. The inclusion criteria were clinically suspected infection on presentation to an emergency department and at least two systemic inflammatory response syndrome signs, not including high leukocyte counts. We excluded high leukocyte counts as a criterion because the result of the blood sample in many cases would not be available for the clinicians when they do their initial judgment of severity of the patient’s condition.

Organ failure was defined as fulfilling one of the following criteria at arrival to the emergency department: oxygen saturation <90% or PaO2/FiO2 <40 kPa, altered mental status, urine output <0.5 mL/kg/hour or increase in serum creatinine >50 micro mol/L, international normalized ratio >1.5 or activated partial thromboplastin time > 60 seconds, platelet count < 100 or 50% reduction in previous three days, serum bilirubin >70 mmol/L, serum lactate >4 mmol/L, blood pressure <90 systolic, mean arterial pressure <60, or fall in mean arterial pressure >40 mm Hg. We did not use a Sequential Organ Failure Assessment (SOFA) score for inclusion, as this was not in use at the emergency departments, and it was not possible to collect data retrospectively in order to evaluate the patients’ SOFA score.

### Data collection

We used a two-step case ascertainment approach to identify eligible patients. First, we searched the Norwegian Patient Registry using a predefined list of the ICD-10 diagnostic codes that are most commonly used in Norway to classify sepsis and infections [18] (see S1 File). The patient registry contains diagnostic and therapeutic codes for all hospital admissions. The search produced a list of patients who had been discharged from the participating hospitals with a sepsis and/or infection code, together with an identification number that enabled access to the corresponding health records. Second, information about the patients’ clinical status upon presentation to the emergency department from the individual patient records was assessed on-site at the hospitals to determine eligibility. Out of 5188 patients initially screened for eligibility, 1559 patients were included in the study (see S1 Fig). The sample size was arrived at through power calculations to detect changes between the baseline measurements (which is the study sample used for this study) and post-inspection measurements. The power calculations are explained in detail in the study protocol [15].

Six regional inspection teams from the County Governors, who carry out inspections on behalf of the Norwegian Board of Health Supervision, were tasked with assessing eligibility and collecting data. The inspections were headed by experienced team leaders with particular training in performing similar inspections. The teams consisted of a minimum of four inspectors with medical and legal expertise, including an independent senior consultant physician in internal medicine or critical care medicine.

Each team performed four inspections within a time frame of about seven weeks in four geographically proximate hospitals. The inspections were rolled out sequentially from March 2016 to February 2017, averaging two per month. The sequence of inspections was randomized to facilitate comparison of outcomes before and after the inspections. The details of and rationale for the study design are described in the published study protocol [15].

The teams collected the data retrospectively during their inspection site visits. To allow for possible changes in clinical performance over time, they sampled data from two different time intervals for each hospital. For each time interval, we aimed to include the last 33 consecutive patients with sepsis who fulfilled the inclusion criteria on presentation to the emergency departments. The first sample included the last 33 patients admitted at each hospital before 1 October 2015, which was immediately before the Norwegian Board of Health Supervision announced the inspection campaign. The second sample included the last 33 patients before inspection of each hospital.

Data were recorded manually on case record forms, and subsequently digitized and saved to a single database containing information from all 24 participating hospitals. Upon completion of the database, we obtained information from the National Patient Registry on 30 day all-cause mortality and Charlson Comorbidity Index [19] for all patients, based on their national identity number and date of hospital admittance.

### Data analyses

To assess the timeliness of diagnostic procedures, we calculated the percentages and 95% confidence intervals of patients with sepsis who had been documented as undergoing diagnostic procedures and receiving antibiotics within specified time limits (see Box 1). We did the analysis with all patients included and for the subgroup of patients with organ failure. Because several patient records lacked data on one or more measures of care delivery, we have also provided the number of records with missing documentation.

To estimate mean difference in time to first dose of antibiotics between categories of clinical procedure variables, we performed linear regression analyses using minutes to first dose of antibiotics as the outcome variable. Following previous research [16] and knowledge of the clinical care process in emergency departments, we focused on the following clinical procedures as exposure variables: triage within 15 minutes, examination by a physician in accordance with priority ascertained by triage, blood lactate measurements within one hour, and evidence of an adequate observation regimen within the emergency department. We performed univariate analyses for each procedure and then included all factors in a multivariable analysis.

Analyzing the association between time to diagnostic procedures and time to treatment, we needed to address the question of whether all kinds of delays were the results of clinical decisions to prioritize care for the patients who had the most serious clinical condition, thus making any association between delays to diagnostic procedures and delays to treatment a spurious one. We controlled for such confounding by indication by including age, organ failure, and comorbidity as covariates in adjusted analyses. The results were then checked against a subgroup analysis of patients who had a serious medical condition at arrival identified by a red or orange triage color (see S2 File). We made additional adjustment for elapsed time since commencement of the study. Elapsed time was measured using calendar days, and was added as a cubic term.

We used a logistic regression model to estimate the 30-day all-cause mortality rate in relation to time to antibiotic administration. In this analysis, we used all-cause mortality as the binary outcome variable and time to first dose of antibiotics as a cubic exposure term, allowing for a non-linear relationship. We also made adjustment for age, year of admission (entered as categorical variables), Charlson comorbidity index, and organ failure as adjustment variables. We present the model-predicted mortality rates by time to antibiotic administration in a graphical format.

Patients who either had no antibiotic indication or had received antibiotic treatment before admittance in the emergency department were excluded from the regression analyses. So were patients for whom we lacked information on time to antibiotic treatment.

For some patients, data were missing for one or several of the variables included as covariates in the analyses. The results from blood samples are imported to the electronic health record; we therefore coded blood lactate as not taken within one hour when it was not documented in the patient record. Similarly, we coded patients who lacked documentation on adequate observation regimen within the emergency department as not having been observed adequately. We imputed data for four variables in our data set: time to antibiotics in minutes, time to examination by a physician, time to triage, and organ failure. Missing values were imputed using fully conditional specification [20]. See S2 File for more information about the treatment of missing data and the regression models.

The regression analyses were first performed including the whole study sample, and then for the sub-group of patients with organ failure (see supplemental S3 File).

We performed the statistical analyses with *Stata* versions SE 15.1 and IC 16.0 (StataCorp LP, College Station, TX, USA). For all regression models, we obtained cluster-robust standard errors of model parameters to account for intra-cluster correlations.

## Results

The study included 1559 patients from 24 Norwegian hospitals from all the 19 Norwegian counties. Table 1 shows characteristics of the study cohort.

**Table 1:** Selected characteristics of the study cohort.

	Male	Female	All
N	800 (51.3%)	759 (48.7%)	1559
Mean (standard deviation) age	69.3 (16.5)	64.6 (20.9)	67.0 (18.9)
Median (min—max) age	72 (18–98)	69 (18–99)	71 (18–99)
Mean (standard deviation) CCI*	3.0 (2.5)	2.2 (2.2)	2.6 (2.4)
Organ failure	313 (39.5%)	244 (32.8%)	557 (36.3%)

* Charlson Comorbidity Index

The percentages of patients who received care in line with the pre-defined standards are shown in Table 2.

**Table 2:** Proportion of patients who underwent clinical procedures and received treatment in line with pre-defined standards.

			Number of patient records		Percent of patients documented receiving recommended care (95% confidence interval)	
Measure			Records with documentataion*	Records lacking documentation	All study patients^†^	Patients with organ dysfunction
Diagnostics						** **
	Complete assessment of vital signs within 1 hour		1360	199	83.6 (81.8 to 85.5)	81.0 (77.7 to 84.2)
	Pulse rate measured within 1 hour		1496	63	93.3 (92.9 to 94.6)	94.1 (92.1 to 96.0)
	Temperature measured within 1 hour		1492	67	93.1 (91.9 to 94.4)	94.3 (92.3 to 96.2)
	Blood pressure measured within 1 hour		1493	66	92.7 (91.9 to 94.0)	93.9 (91.9 to 95.9)
	Respiration rate measured within 1 hour		1476	83	91.5 (90.9 to 92.9)	93.4 (91.3 to 95.4)
	Mental status assessed within 1 hour		1390	169	86.0 (84.8 to 87.7)	83.1 (80.0 to 86.2)
	Blood culture taken prior to administration of antibiotics		1350	95	85.3 (83.8 to 87.1)	84.6 (81.6 to 87.6)
	Adequate supplementary examinations to identify source of infection		1548	11	93.7 (92.9 to 94.9)	93.7 (91.7 to 95.7)
	Time to triage (≤ 15 min)		1375	184	72.9 (70.7 to 75.1)	77.0 (73.5 to 80.5)
	Adequate observation regimen in ED		1524	35	44.4 (41.4 to 46.9)	47.4 (43.2 to 51.6)
	Examination by physician in accordance with triage urgency		1105	454	44.9 (42.4 to 47.4)	47.6 (43.4 to 51.7)
	Leukocytes count	Blood samples taken within 1 hour	1534	25	87.1 (85.8 to 88.8)	88.5 (85.9 to 91.2)
	Hemoglobin		1533	26	87.2 (85.8 to 88.8)	88.2 (85.5 to 90.8)
	C-reactive protein		1534	25	87.0 (85.8 to 88.7)	88.5 (85.9 to 91.2)
	Creatinine		1525	34	86.7 (85.8 to 88.3)	88.3 (85.7 to 91.0)
	Electrolytes		1524	35	86.8 (85.8 to 88.5)	88.3 (85.7 to 91.0)
	Platelet count		1510	49	85.8 (84.8 to 87.5)	87.4 (84.7 to 90.2)
	Glucose		1492	67	85.1 (83.8 to 86.9)	87.6 (84.9 to 90.4)
	Bilirubin		963	482	62.0 (59.6 to 64.4)	66.2 (62.3 to 70.2)
	Blood lactate		955	604	48.6 (46.4 to 51.1)	58.5 (54.4 to 62.6)
Treatment						
	Antibiotics within 1 hour^‡^		1313	132	25.5 (23.2 to 27.7)	30.4 (26.4 to 34.3)
	Antibiotics within 2 hours^‡^		1313	132	55.5 (52.9 to 58.1)	59.4 (55.2 to 63.6)
	Antibiotics within 4 hours^‡^		1313	132	79.7 (77.6 to 81.7)	82.5 (79.2 to 85.7)

* Total number of records: 1559

^†^ Patients with suspected infection together with two systemic inflammatory response syndrome signs

^‡^ n = 1438 (patients registered as needing antibiotic treatment and not having received antibiotics prior to admission)

Of the patients in our sample, 72.9% (95% confidence interval 70.7 to 75.1), had documented triage within 15 minutes of presentation to the emergency department, 44.9% (42.4 to 47.4) were examined by a physician in accordance with the priority specified during triage, and 83.6% (81.8 to 85.5) had a complete set of vital signs recorded within one hour of presentation. Blood samples were obtained within one hour from more than 80% of the patients for all specified tests except for bilirubin and lactate, 62.0% (59.6 to 64.4) and 48.6% (46.4 to 51.1), respectively; 44.4% (42.4 to 47.4) were adequately observed while in the emergency department according to the degree of priority assigned during triage; and 25.4% (23.2 to 27.7) and 55.5% (52.5 to 58.0) of the patients received antibiotics within one and two hours, respectively.

We found an association between non-completed or delayed diagnostic procedures and prolonged time to administration of antibiotics. In adjusted analyses (Model 1 in Table 3), patients who had not been triaged within 15 minutes had in average an extra delay of 54.7 minutes (95% confidence interval 33.2 to 76.2) to administration of antibiotics. We found a similar pattern of prolonged time to administration of antibiotics of cases where patients were not examined by a physician within the time limits set in triage, 61.2 minutes (40.8 to 81.6), not having blood lactate measured within one hour, 86.2 minutes (71.5 to 100.8), and not having an adequate observation regimen, 39.3 minutes (21.8 to 56.8). When we included all four procedures in one regression analysis (Model 2 in Table 3), they together predicted a delay of 159 minutes to first dose of antibiotics.

**Table 3:** Linear regression for factors associated with delay in antibiotic treatment.

	Unadjusted	Model 1*	Model 2†
	*b (95% CI)*	*b (95% CI)*	*b (95% CI)*
Not triaged within 15 minutes	54.4 (32.9 to 75.9)	54.7 (33.2 to 76.2)	25.8 (3.8 to 47.8)
Examination by physician not in accordance with priority	60.0 (39.2 to 80.9)	61.2 (40.8 to 81.6)	38.0 (16.1 to 59.8)
Lactate not measured within 1 hour	81.6 (65.9 to 97.2)	86.2 (71.5 to 100.8)	71.4 (56.0 to 86.8)
Inadequate observation regimen	41.3 (22.3 to 60.4)	39.3 (21.8 to 56.8)	23.9 (10.5 to 37.3)

Outcome variable: Time to antibiotics measured in minutes. n = 1307

* Adjusted for organ failure, patient age, comorbidity, and time to admission

^†^ Adjusted for the other variables in this table, and organ failure, age, comorbidity, and time to admission

Replicating the regression analyses for the sub-group of patients with organ failure yielded similar results, with the model including all four factors also predicting an extra delay of 159 minutes for patients with organ failure (see S3 File).

**Fig 1:**
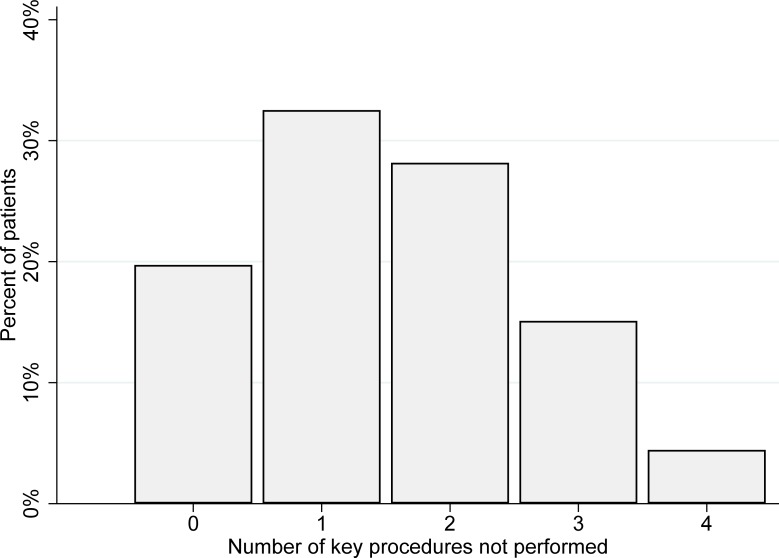
Distribution of patients according to number of non-completed or delayed key diagnostic procedures. Key procedures: triage within 15 minutes, examination by physician in accordance with urgency specified during triage, blood lactate measured within 1 hour, adequate observation regimen. N = 1559.

Fig 1 shows the distribution of patients according to the number of the four specified procedures that were not performed within the recommended time limits.

The 30-day all-cause mortality was 9.9% (8.4 to 11.4) for the entire study sample and 17.4% (14.2 to 20.5) for patients with documented organ failure.

**Fig 2:**
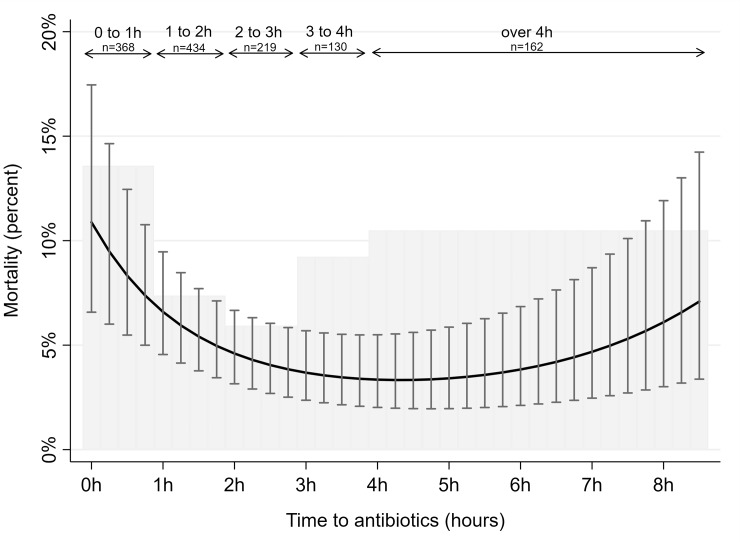
All-cause 30-day mortality by time to antibiotic treatment. Gray shaded histogram represents mortality rates according to time to antibiotic treatment in hours. Solid black curve with bars represents model-predicted mortality rates with 95% confidence intervals according to time to antibiotic treatment in minutes using logistic regression models, adjusted for patient’s age, date of admission, comorbidity, and presence of organ failure. Date of admission was measured using calendar days since study start, entered as a polynomial function with first (b -0.011 p<0.001), second (b 2.5e-5 p<0.001) and third degree (b -1.2e-8 p<0.01) variables. The model prediction uses average values for adjustment values.

Fig 2 displays the observed 30-day all-cause mortality in hourly intervals for time from admission to administration of antibiotics (bars). Patients receiving antibiotics within 1 hour had an observed mortality of 13.6% (10.1 to 17.1), whereas those receiving antibiotics in the timespan 2 to 3 hours after admission had an observed mortality of 5.9% (2.8 to 9.1) and those receiving antibiotics 4 hours or later after admission had an observed mortality of 10.5% (5.7 to 15.3).

Fig 2 also shows the model-predicted 30-day all-cause mortality according to time to antibiotic treatment in minutes, adjusted for patient’s age, date of admission and presence of organ failure (shown by the solid black curve).

Replicating the analysis of the association between time to antibiotic treatment and 30-day mortality for the sub-group of patients with organ failure, we found a similar curvilinear trend where predicted mortality was highest for patients who received antibiotics within one hour (see S3 File).

## Discussion

### Principal findings

In this study of 24 emergency departments, we found that they frequently failed to perform important diagnostic procedures in time, and that delays in or non-completion of diagnostic procedures were associated with prolonged time to administration of antibiotics. In 46% of the study patients, two or more of the following four key procedures had not been carried out in a timely manner: triage within 15 minutes, examination by physician in accordance with priority as set in triage, measuring blood lactate within one hour, and adequate observation. Non-completion or delay of these procedures together predicted a delay of 159 minutes to administration of antibiotics. We also found a substantial variation in mortality according to time to antibiotics. Patients who started antibiotic treatment between 2 and 3 hours after admission had lower mortality than those who started antibiotics earlier or later.

### Strengths and limitations

The main strengths of our study are the combination of inclusion procedures and the size of the patient cohort from 24 different study sites. The hospitals that the study patients attended can be considered representative of Norwegian hospitals because they included all university and regional hospitals and a geographically based selection of local hospitals, serving 75% of the total Norwegian population. Moreover, we included consecutive patients admitted to the emergency departments during two different time periods by using a cluster randomized sampling approach. Therefore, our study cohort is representative of patients admitted to Norwegian emergency departments with infection and meeting two or more systemic inflammatory response syndrome criteria.

Another important strength of our study is that we manually reviewed all patient records and established eligibility on the basis of recorded clinical data rather than on diagnostic codes alone. The latter approach can cause ascertainment bias and misleading inferences because coding practices for sepsis can vary over time and between hospitals [21].

A limitation of our study is the use of systematic inflammatory response syndrome (SIRS) as an inclusion criterion, in line with the *Sepsis-2* definition. Since the protocol was initially drafted and the project started, a *Sepsis-3* definition as a *life-threatening organ dysfunction caused by a dysregulated host response to infection* was proposed [6]. As compared to Sepsis-2, Sepsis-3 represents only a minority of patients with infection [22]. Thus, our findings cannot be directly generalized to patient groups that have sepsis according to the Sepsis-3 definition. We have performed sub analyses of patients with organ failure, which is a group of patients that more closely overlaps with the Sepsis-3 definition, to make it easier to compare our findings to those of studies relying on the Sepsis-3 definition. These analyses show similar results for the sub-group of patients with organ failure as those of the whole study sample.

Another limitation of our study is that we did not have data on severity of sepsis in the form of commonly used severity scores like SAPS 2 (simplified acute physiology score) or APACHE II (acute physiology and chronic health evaluation), or a detailed organ failure assessment score like SOFA (sequential organ failure assessment). We did control for age, presence of organ failure, and comorbidity, which are three important variables associated with severity of sepsis [23]; however, even when controlling for these variables, the associations we found between time to antibiotics and mortality were probably subject to confounding by unmeasured variations in severity of illness and patient characteristics. As such, the study design does not allow for unbiased estimation of treatment effects.

### Comparison with other studies

The delays we identified in diagnostic procedures are consistent with previous research findings of delay in time to triage [24], recording of vital signs [5, 7], measurement of blood lactate [25, 26], and delays in recognition of sepsis in general [7]. The processes we found to be lacking are essential for reaching an accurate diagnosis and institution of treatment in a timely manner [27].

We found that 25.5% and 55.5% of patients received antibiotics within one and two hours, respectively. This is in line with or slightly faster than the timing reported in previous studies with comparable patient cohorts in emergency department settings, which found that 28% of patients received antibiotics within 1 hour [7] and that median times to commencing antibiotics were 2.1 hours [28] and 182 minutes [29].

No previous studies have demonstrated the extent of delay or non-completion of diagnostic procedures for patients with sepsis in a large, representative cohort of patients admitted to emergency departments, or assessed the association between non-completed or delayed procedures and prolonged time to antibiotic administration.

The mortality rate in our study was in line with mortality rates reported in previous research in an emergency department setting [11]. However, we found a curvilinear association, where patients receiving early treatment and treatment later than four hours after admission had higher mortality rates than those receiving treatment between two and four hours after admission. This parabolic trend conflicts with a previous report of a linear increase in mortality with increasing time to antibiotics [13].

### Interpretation of findings and implications

There is an ongoing debate concerning how timing of antibiotics for patients with sepsis should be operationalized in guidelines. The guidelines have come under criticism for not being adequately based in empirical evidence and being overly reliant on treatment protocols mandating antibiotic initiation within one hour of triage [30] and early administration of broad-spectrum antibiotics to all patients with sepsis [31]. Commenting on the sepsis guidelines, the Infectious Disease Society of America recommends administration of antibiotics as soon as possible to patients with severe infections. However, they warn that rigid guideline recommendations with fixed time frames might increase the likelihood of broad-spectrum antibiotics will be given to uninfected patients [32]. In line with this argument, we maintain that the timing of antibiotic treatment should be an informed clinical decision rather than a consequence of unintended delays in diagnostic procedures. Our study indicates that the latter might often be the case: Delays in diagnostic procedures are common and they might lead to delayed treatment.

Emergency departments must therefore attend to optimizing diagnostic screening to improve time to treatment and overall management of sepsis. The Surviving Sepsis Campaign recommends a performance improvement program that includes screening for sepsis [33]; however, it is still necessary to define more precisely what screening measures should be implemented and how they should be monitored as part of the improvement program. We argue that our findings can inform this work.

We assert that the non-linear association we found between antibiotic treatment and mortality reflects the fact that many patients with sepsis are already critically ill when they present to an emergency department and that these patients are more easily recognized and given aggressive treatment earlier. This is an observation study, and the associations we found between time to antibiotics and mortality were probably subject to confounding by unmeasured variations in severity of illness and patient characteristics. Thus, one should not draw conclusions regarding the efficiency of antibiotic treatment at specific time intervals based on these analyses.

To our knowledge, this is the first multicenter study that assesses the association between a wide array of diagnostic procedures and antibiotic treatment. Previous research, mostly based on single case studies and smaller patient cohorts, has found delays in time to treatment comparable to those we found, suggesting that emergency departments elsewhere in Europe and the USA face challenges regarding variability of performance of initial screening procedures to detect sepsis. We therefore argue that our findings might have relevance for emergency departments outside of Norway.

## Conclusions

We found that key procedures for recognizing sepsis and organ failure in the emergency department were delayed or not carried out in a substantial proportion of patients with sepsis. Delay or non-completion of key diagnostic procedures together predicted a delay of 2.5 hours to the first dose of antibiotics. Initiation of antibiotic treatment should be an informed clinical decision. Delays in antibiotic treatment could potentially have a negative effect on patient outcomes. These findings have important implications for managers and health professionals. The extent of delay and non-completion of important diagnostic procedures suggests that there is a need for systematic improvement efforts in the initial management of patients with sepsis presenting to emergency departments.

## Supporting information

S1 File ICD 10 codes. List of ICD 10 codes used to search the National Patient Register.
(PDF)

S2 File Statistical analyses. Description of how missing data are treated and of how we fitted the regression models.
(PDF)

S3 File Sub-analyses. Sub-analyses of association between diagnostic measures and time to treatment and between time to treatment and mortality for the sub-group of patients with organ failure.
(PDF)

S1 Fig Data collection process. Description Patient flow diagram showing the number of patients included and excluded.
(TIF)
