# Correction: Early diagnosis of sepsis in emergency departments, time to treatment, and association with mortality: An observational study

**DOI:** 10.1371/journal.pone.0248879

**Published:** 2021-03-15

**Authors:** Gunnar Husabø, Roy M. Nilsen, Hans Flaatten, Erik Solligård, Jan C. Frich, Gunnar T. Bondevik, Geir S. Braut, Kieran Walshe, Stig Harthug, Einar Hovlid

There is an error in the caption for Fig 2, “All-cause 30-day mortality by time to antibiotic treatment.” Date of admission was not measured using calendar days and it was not entered as a polynomial function. Date of admission was entered as year, and it was time to antibiotics (measured in minutes), that was entered as a polynomial function. Please see the complete, correct Fig 2 caption here.

**Fig 2:**
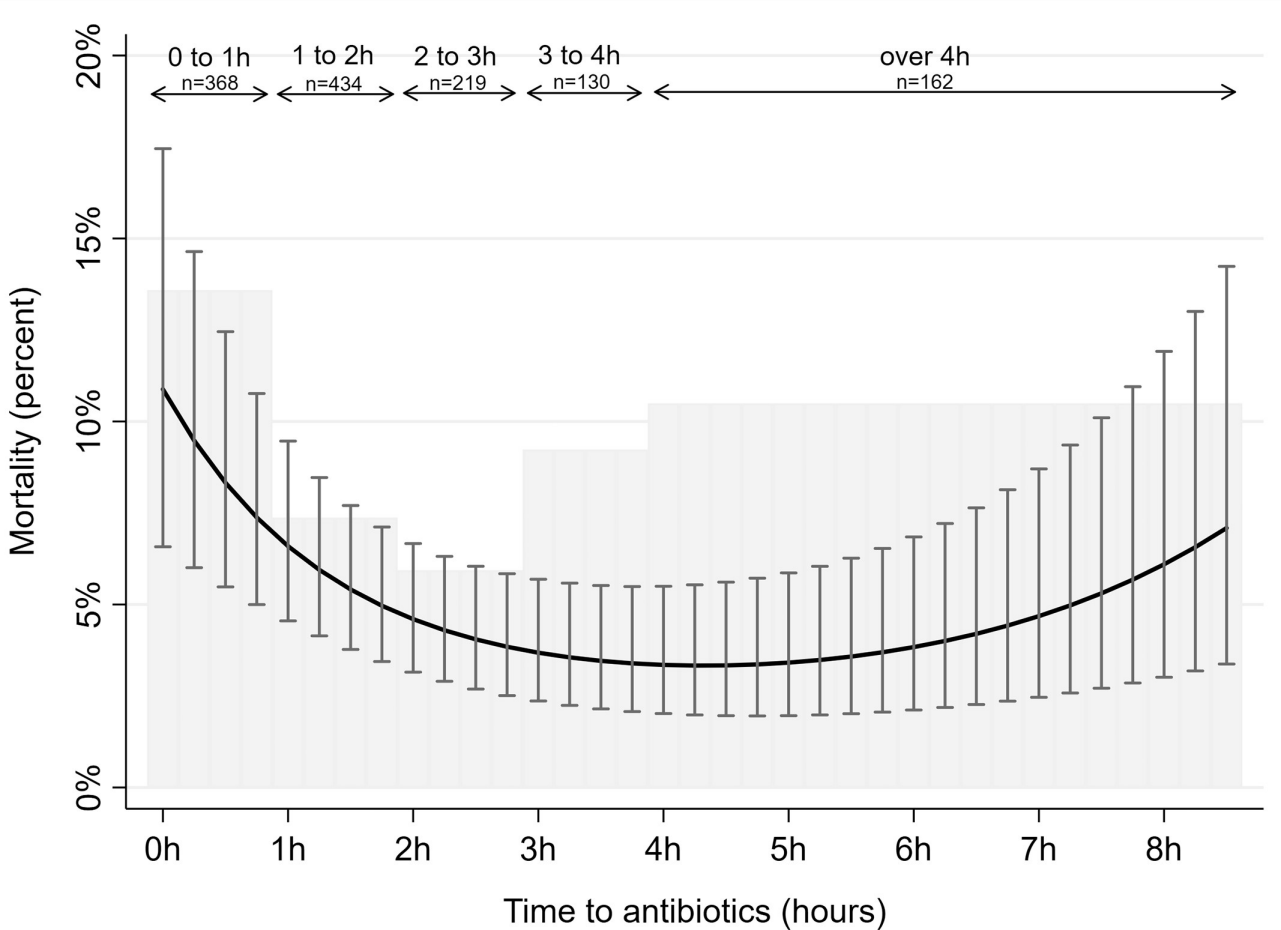
All-cause 30-day mortality by time to antibiotic treatment. Gray shaded histogram represents mortality rates according to time to antibiotic treatment in hours. Solid black curve with bars represents model-predicted mortality rates with 95% confidence intervals according to time to antibiotic treatment in minutes using logistic regression models, adjusted for patient’s age, year of admission, comorbidity, and presence of organ failure. Time to antibiotics was measured in minutes, entered as a polynomial function with first (b -0.011 p<0.001), second (b 2.5e-5 p<0.001) and third degree (b -1.2e-8 p<0.01) variables. The model prediction uses average values for adjustment values.

The same error is repeated in the caption for Fig S3.1 in S1 File, which uses the same type of figure for a sub analysis. The complete, correct Fig S3.1 caption is: Gray shaded histogram represents mortality rates according to time to antibiotic treatment in hours. Solid black curve with bars represents model-predicted mortality rates with 95% confidence intervals according to time to antibiotic treatment in minutes using logistic regression models, adjusted for patient’s age, year of admission, comorbidity, and presence of organ failure. Time to antibiotics was measured in minutes, entered as a polynomial function with first (b -.0113 p < .01), second (b 2.8e-5 p < .05) and third degree (b -1.37e-8 p < 0.1) variables. The model prediction uses average values for adjustment values. N = 488.

Please view the correct S1 File below.

## Supporting information

S1 File Sub-analyses. Sub-analyses of association between diagnostic measures and time to treatment and between time to treatment and mortality for the sub-group of patients with organ failure.
(PDF)
