# Supplementary material for: Early diagnosis of sepsis in emergency departments, time to treatment, and association with mortality: An observational study
Source: PLoS One. 2020 Jan 22;15(1):e0227652. doi: 10.1371/journal.pone.0227652 (PMC6975530; doi:10.1371/journal.pone.0227652)
Supplement: S1 File — List of ICD 10 codes used to search the National Patient Register. (PDF) [file pone.0227652.s001.pdf]

ICD 10 codes used to search the National Patient Register

A02.1 Salmonella sepsis

A20.7 Septicemic plague

A21.7 Generalized tularemia

A22.7 Anthrax sepsis

A24.1 Acute and fulminating melioidosis

A26.7 Erysipelothrix sepsis

A32.7 Listerial sepsis

A39.2 Acute meningococcemia

A39.3 Chronic meningococcemia

A39.4 Meningococcemia, unspecified

A40 Streptococcal sepsis (0.,1.,2.,3.,8.,9.)

A41 Other sepsis (0.,1.,2.,3.,4.,5.,8.,9.)

A42.7 Actinomycotic sepsis

A46 Erysipelas

A48.3 Toxic shock syndrome

A54.8 Other gonococcal infections

O85 Puerperal sepsis

B00.7 Disseminated herpesviral disease

J09 Influenza due to certain identified influenza viruses

J10 Influenza due to other identified influenza virus

J13 Pneumonia due to Streptococcus pneumoniae

J14 Pneumonia due to Hemophilus influenzae

J15 Bacterial pneumonia, not elsewhere classified

J36 Peritonsillar abscess

J39 Other diseases of upper respiratory tract

J85 Abscess of lung and mediastinum

J86 Pyothorax

K65 Peritonitis

K81 Cholecystitis

M72.6 Necrotizing fasciitis

N10 Acute pyelonephritis

R57 Shock, not elsewhere classified

R65 Symptoms and signs specifically associated with systemic inflammation and infection
