# Supplementary material for: Early diagnosis of sepsis in emergency departments, time to treatment, and association with mortality: An observational study
Source: PLoS One. 2020 Jan 22;15(1):e0227652. doi: 10.1371/journal.pone.0227652 (PMC6975530; doi:10.1371/journal.pone.0227652)
Supplement: S2 File — Description of how missing data are treated and of how we fitted the regression models. (PDF) [file pone.0227652.s002.pdf]

Missing data and imputation

Several of the variables in our data set had missing values. The teams that collected data were meticulous in their recording of data from the original patient records, and they would routinely verify that they had included all available data from the patient records. Hence, missing data were indicative of the information never being included in the patient record, rather than failure to collect a complete set of data from the records.

Time to administration of antibiotics had been registered in two separate variables, both in minutes and in hours (measured as prior to admission, within 1 hour, between 1 and 2, between 2 and 3, between 3 and 4, and after more than 4 hours). Time to antibiotic administration measured in hours had fewer missing observations (11%) than time to antibiotic administration measured in minutes (42%), and no observations that had information on minutes to antibiotic treatment lacked information on hours to antibiotics. For the regression analyses, we chose to exclude the observations that had no information on antibiotic treatment. We also excluded observations where antibiotics were administered prior to admittance (5%), or where the patients' records indicated that they should not receive antibiotic treatment (2%). In table 2 in the article, these two last groups were subtracted from total n when reporting the proportion of patients receiving antibiotic treatment. The total n included, however, patients with missing data on time to antibiotic treatment.

The missing data on the other variables used in the regression analyses could potentially lead to bias and loss of power in the analyses. To alleviate these problems, we decided on a strategy of combining recoding and multiple imputation of missing data.

For two of the diagnostic process variables that had missing data in the original data material, blood lactate and adequate observation regimen, we coded missing values as 0, i.e. incomplete performance of the process. We did this because information on these procedures

should be documented in the patient record. Lack of documentation therefore indicated that the procedures were not completed according to guidelines.

For four variables, we imputed missing data: Time to antibiotics in minutes (42% missing), time to examination by a physician (35% missing), time to triage (12% missing), and organ failure (3% missing). Imputation is recommended in cases where data is *missing at random* (MAR). Data are MAR when missingness in the relevant variable only depends on data from variables that we have observed and can control for. Assuming missing data to be MAR, we created 100 imputed datasets by using the fully conditional specification algorithm [1], a method for running a series of regression analyses, one for each variable to be imputed. To obtain pooled estimates with 95% confidence intervals across the imputed datasets, we used Rubin's combination rules as implemented in the *mi suite of commands* in Stata.

We chose the imputation model according to the distribution of the variable to be imputed. For our primary variable of interest for the regression analyses, time to antibiotic administration in minutes, we used interval regression in the imputation model. Upper and lower bounds for imputed values were set based on information about time to antibiotic administration in hours. We imputed the other three variables using logit models. Each imputation model included the other imputation variables as predictor variables, along with the rest of the variables which were to be used in the regression analyses: age, admission year, Charlson comorbidity index, blood lactate measured within one hour, adequate observation regimen, all-cause mortality after 30 days. In addition, we added hours to antibiotic treatment as an auxiliary variable.

Fitting the linear model

We performed sensitivity tests by omitting some of the most extreme values for time to treatment with antibiotics; these were six observations which had more than 1000 minutes from admission to administration of antibiotics. This adjustment proved to have an impact on model fit and individual coefficient estimates, so we chose to omit these six outliers in the final models.

In addition to testing the models with and without the outlier variables, we also tested modeling the analyses using standard Poisson and Negative binomial regression, as well as fitting an identical linear regression model using a log transformed outcome variable. The log transformed model had slightly better fit than the linear model without the six extreme values, but the two models showed similar effect sizes and p-values. We chose to report the results of the linear analysis in the paper because the linear model provides predictions of delay in minutes for each predictor variable, rather than coefficients that need to be exponentiated to provide information on delay in relative terms.

In all analyses, we first analyzed the crude association between outcome and exposure variables, and them we ran the analyzes again, controlling for age, organ failure, comorbidity, and time to admission. We also performed sensitivity analyses by running the regression analyses for the subgroup of patients who were triaged to code red or orange, i.e., a subgroup of patients who needed expedient attention. This analysis found that, when controlling for the other potential confounders, the associations between timely diagnostic tests and time to antibiotic treatment in the subgroup of patients triaged as orange or red were similar to the associations we found in the patient group as a whole.

Estimates from logistic regression

After fitting the logistic regression model, we obtained predictive margins with confidence intervals using the user written Stata command *mimrgns* [2], requesting conditional predictions for each 15 minutes from zero to nine hours. We re-calculated these margins into predictions of probabilities for outcome 1 (death within 30 days) using logit transformation. Subsequently, we imported the recalculated predictive margins into Stata, along with mean mortality rates by hour to antibiotics. We then combined a bar graph for the empirical mortality rates and a line graph for the predicted probabilities into one figure, using Stata's *twoway* command.

References

1. Buuren S. Flexible Imputation of Missing Data, Second Edition: Chapman & Hall/CRC; 2018.
2. Daniel K. MIMRGNS: Stata module to run margins after mi estimate. S457795 ed: Boston College Department of Economics; 2014.
