# Supplementary figures and images for: Early diagnosis of sepsis in emergency departments, time to treatment, and association with mortality: An observational study
Source: PLoS One. 2020 Jan 22;15(1):e0227652. doi: 10.1371/journal.pone.0227652 (PMC6975530; doi:10.1371/journal.pone.0227652)

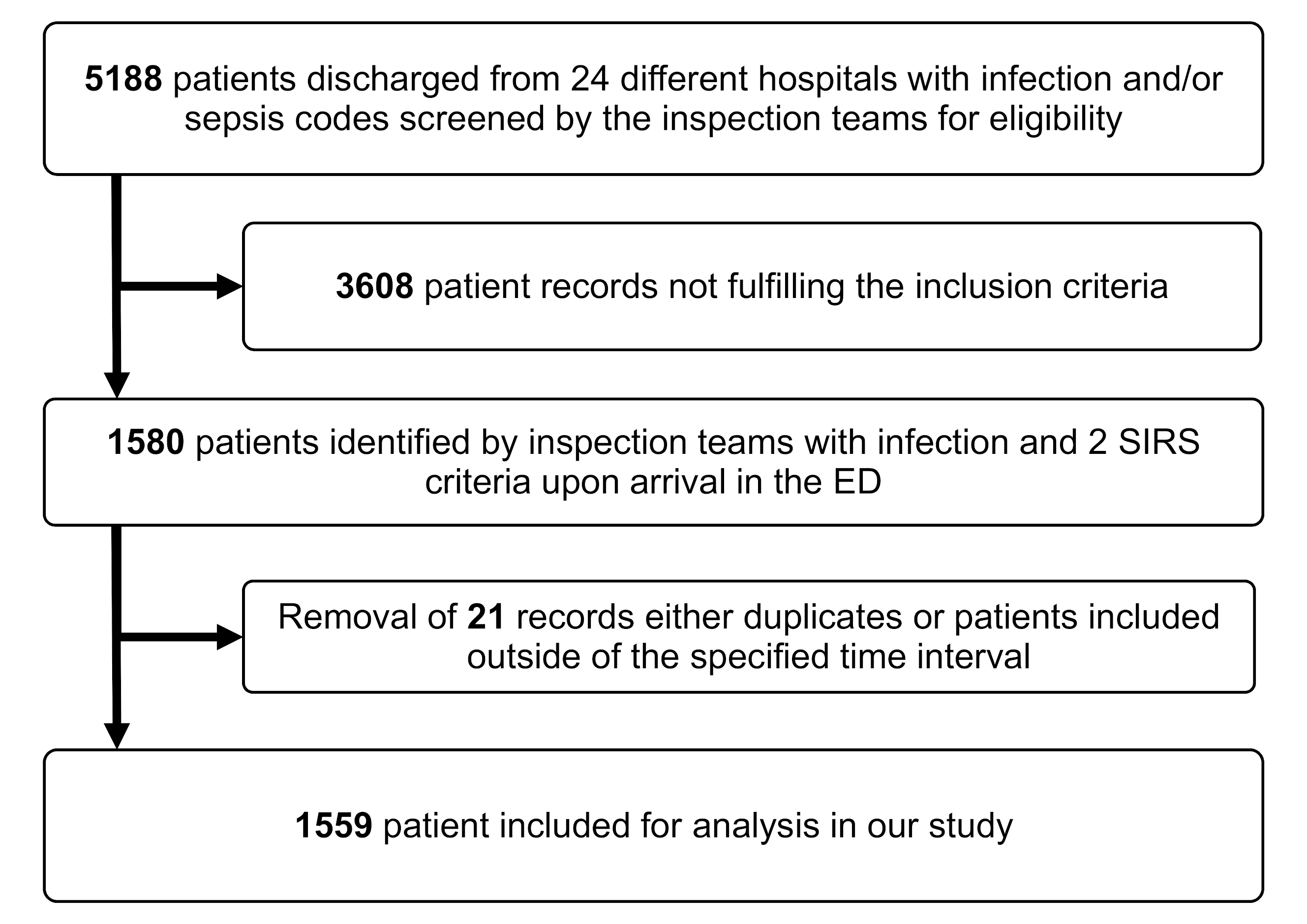

Supplement: S1 Fig — Description Patient flow diagram showing the number of patients included and excluded. (TIF) [file pone.0227652.s004.tif]
