# Supplementary material for: Correction: Early diagnosis of sepsis in emergency departments, time to treatment, and association with mortality: An observational study
Source: PLoS One. 2021 Mar 15;16(3):e0248879. doi: 10.1371/journal.pone.0248879 (PMC7959348; doi:10.1371/journal.pone.0248879)
Supplement: S1 File — Sub-analyses of association between diagnostic measures and time to treatment and between time to treatment and mortality for the sub-group of patients with organ failure. (PDF) [file pone.0248879.s001.pdf]

Sub-analyses – patients with organ failure

We replicated the analyses of association between diagnostic procedures and time to treatment and between time to treatment and mortality on the sub-group of patients with organ dysfunction (Table S3.1 and Fig S3.1, below).

Table S3.1. Linear regression for factors associated with delay in antibiotic treatment. Patients with organ failure.

	**Unadjusted** *b (95% CI)*	**Model 1*** *b (95% CI)*	**Model 2†** *b (95% CI)*
Not triaged within 15 minutes	53.5 (21.4 to 85.7)	56.1 (26.2 to 86.0)	17.1 (-11.1 to 45.3)
Examination by physician not in accordance with priority	64.2 (34.8 to 93.5)	67.4 (39.1 to 95.7)	47.2 (18.5 to 75.9)
Lactate not measured within 1 hour	78.5 (56.9 to 100.2)	84.3 (65.2 to 103.3)	25.3 (8.6 to 42.0)
Inadequate observation regimen	42.8 (21.4 to 64.2)	42.3 (22.0 to 62.6)	69.1 (48.6 to 89.5)

Outcome variable: Time to antibiotics measured in minutes. n=487

* Adjusted for patient age, comorbidity, and time to admission

† Adjusted for the other variables in this table, and age, comorbidity, and time to admission

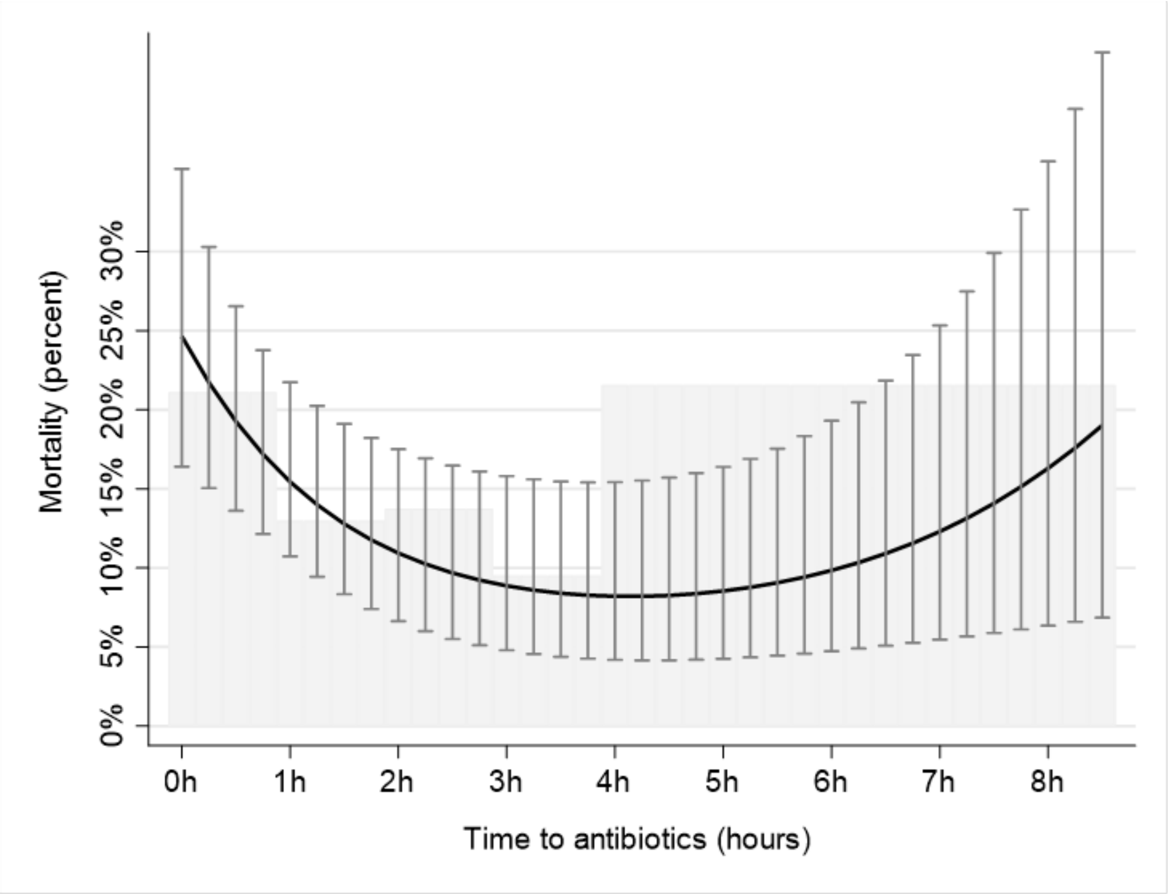

Fig S3.1. All-cause 30-day mortality by time to antibiotic treatment. Patients with organ failure.

Gray shaded histogram represents mortality rates according to time to antibiotic treatment in hours. Solid black curve with bars represents model-predicted mortality rates with 95% confidence intervals according to time to antibiotic treatment in minutes using logistic regression models, adjusted for patient's age, year of admission, comorbidity, and presence of organ failure. Time to antibiotics was measured in minutes, entered as a polynomial function with first (b -.0113 $p < .01$), second (b 2.8e-5 $p < .05$) and third degree (b -1.37e-8 $p < 0.1$) variables. The model prediction uses average values for adjustment values. N=488.
